# Heavy Metal Pollution and Soil Quality Assessment under Different Land Uses in the Red Soil Region, Southern China

**DOI:** 10.3390/ijerph19074125

**Published:** 2022-03-31

**Authors:** Zhiping Yang, Rong Zhang, Hongying Li, Xiaoyuan Zhao, Xiaojie Liu

**Affiliations:** 1 Jiangxi Research Academy of Ecological Civilization, Nanchang 330036, China; yangzhiping8998@163.com; 2 Technical Centre for Soil, Agriculture and Rural Ecology and Environment, Ministry of Ecology and Environment, Beijing 100012, China; zhangrong@tcare-mee.cn; 3 Foreign Environmental Cooperation Center, Ministry of Ecology and Environment, Beijing 100035, China; li.hongying@fecomee.org.cn; 4 Institute of Geographic Sciences and Natural Resources Research, Chinese Academy of Sciences, Beijing 100101, China; zhaoxiaoyuan@cugb.edu.cn

**Keywords:** soil quality assessment, TOPSIS, land use types, positive matrix factorization

## Abstract

The influences of different land uses associated with human activities on soil quality and the redistribution of heavy metal in soil have been widely concerned. Surface soil samples were obtained to assess comprehensive soil quality in a typical red soil region of southern China, combining the heavy metal pollution evaluation with fertility evaluation. It can be learned from the results that the overall level of soil fertility was at medium and lower level, and soil heavy metal pollution risk in the study area in a few regions had reached warning line and slight pollution line, and there was a risk of potential pollution. TOPSIS evaluation results showed that the comprehensive soil quality was mainly good quality and moderate quality, accounting for 31.7% and 29.0% of the total land area, respectively. Positive matrix factorization (PMF) model results showed that transportation source contributes a lot in terms of Cd and Pb. As for Cr, natural source contributes 53.8%. In terms of Cu and Zn, agriculture source contributes 50.7% and 38.7%, respectively. In a word, the comprehensive soil quality assessment in red soil region of southern China provides an important basis for the scientific management and sustainable utilization of soil resources.

## 1. Introduction

Soil heavy metals pollution has gained more and more attention due to its refractory and difficulties in detoxifying biologically [1,2,3]. Heavy metals can be concentrated in the human body through food and respiratory intaking, posing harm to human health. For example, Itai-itai disease is contributed to environmental cadmium (Cd) exposure [4]. Excessive intake of Cu can cause Cu poisoning, acute hemolysis, and renal dysfunction [5]. Pb is one of the most common heavy metal pollutants in soil. Pb has no physiological function to human body and serious harm to human health including slowing down children’s cognitive development and weaken their intelligence [6]. Cr and Zn are recognized as toxic elements that can change the functions of human nervous system and respiratory system and disrupt the endocrine system [7]. In the south of China, agricultural soils were seriously destroyed by heavy metal pollution according to the national communique of soil pollution survey. In addition, human activities such as cutting down trees, and industrial and mining enterprises are regarded as being the catalysts, intensifying the pollution [8].

Due to an increasing population, humans have converted natural areas to agriculture or changed rice fields to economic crop fields to satisfy food needs [9,10]. Some studies have reported that the physicochemical properties of soil could be impacted by different land uses (wetlands, grasslands, and afforestation), which directly or indirectly could influence the geochemical behaviors of heavy metals [11,12]. For example, greenhouse vegetable cultivation may cause soil nutrient enrichment or imbalance [13]. Afforestation changes soil pH and organic matter content and affects soil heavy metal solubility [14]. Wang et al. [15] found that changing from rice/wheat rotation to vegetables can resulted in shallow tilled layer and compaction. Islam and Weil [16] found that land use changes, such as forest degradation and soil cultivation, contributed to soil mechanical composition changing, and some soil chemical indexes content decreased, such as N and microbial biomass C. Their results suggested that land use changes can affect plants, land, and water provision, diminishing soil quality and causing soil contamination. In recent years, more and more studies have focused on assessing soil quality in single land use such as mining area [17], wetland [18], afforestation [19], and cropland [20], but few researchers have focused on different land uses in one study area. Therefore, assessing soil quality under different land uses is extremely important, especially in red soil areas.

Soil quality is an all-round reflection of soil characteristics and also the most sensitive indicator, revealing soil condition dynamics in order to embody the impact of natural factors and human activities on the soil, which can be assessed through physical and chemical properties evaluation [21]. Soil fertility and soil environment quality are the most important indicators in soil quality. At present, there is a lack of a unified evaluation method. Generally, soil environment quality is assessed based on heavy metals using the Single pollution index and the Nemerow composite pollution index [22,23]. Meanwhile, Fuzzy multi-attribute evaluation [24] is conventionally used to assess soil fertility quality based on nutrient elements. In conformity, the chief research objectives were to: (1) combine fuzzy mathematical model with a Nemerow composite pollution index using TOPSIS to assess soil quality comprehensively; (2) analyze the correlation of soil physicochemical properties; (3) classify the overall levels of soil quality based on a comprehensive assessment of soil heavy pollution and soil fertility status; (4) use a positive matrix factorization (PMF) model to find the pollutant source. The study results will provide an important basis for scientifically managing and reasonably using the soil resources.

## 2. Materials and Methods

### 2.1. Study Area

**Figure 1:**
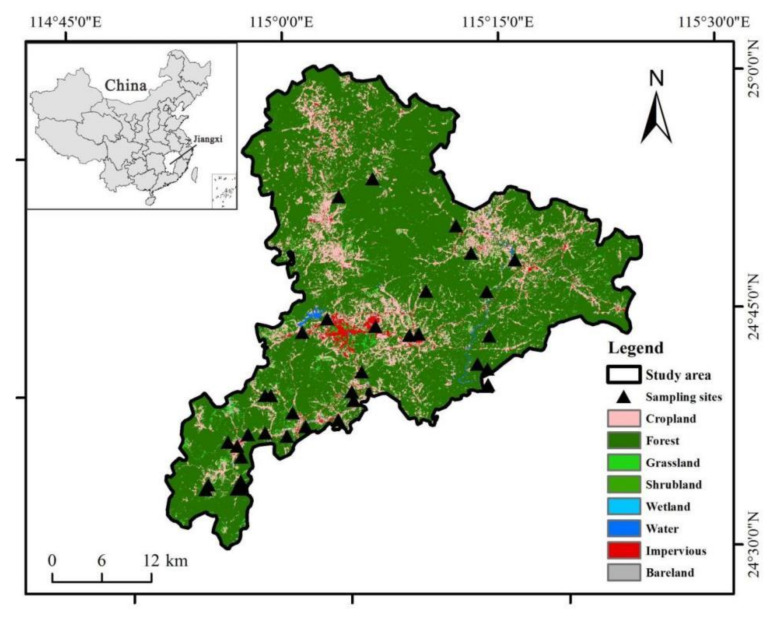
Location of study area and sampling sites.

The study area is situated in Dingnan County (24°26′–25°05′ N, 114°46′–115°23′ E), Jiangxi Province, Southeast China (Figure 1). Jiangxi province is an important base for developing mineral resources in China, especially Ganzhou city has the highest development intensity. Dingnan is an important non-ferrous metal base county in the whole city, the whole province, and even the whole country. The mineral resources, mainly including rare earth and tungsten, are widely distributed, diversified, and large in reserves. However, the early extensive development mode caused serious soil and water pollution, spreading all over 18 counties and urban areas of Ganzhou city. The study area has a central Asia continental tropic monsoon humid climate. The average temperature is 18.8 °C and the annual rainfall is 1600 mm.

### 2.2. Sample Collection and Laboratory Analysis

From different land uses (vegetable, orchard, paddy field, forest, dry land, and grassland) (Figure 1), 43 surface soil (0–20 cm) samples were collected. Fourteen samples came from forest, which was eucalyptus. Five samples came from grassland and five samples came from dry land. Five samples came from vegetable, which was rape. Five samples came from orchard, which was navel orange. The pH of rape and navel orange soil are 5–6, and weak acidity or neutral soil are the most favorable and these crops require good aeration performance of soil, in which loam is the best. Nine samples came from a paddy field, which was harvested twice a year. The soils have not been ploughed and their properties have not changed much. These sampling points were geo-located using global positioning system receivers. Each soil sample was collected with a stainless steel auger to a depth of 0–20 cm and thoroughly mixed with five nearby samples. The soil samples were packed in polyethylene zip bags, then labelled, and transported to the lab. After pretreatment, samples were digested in HNO_3_-HCl-HClO_4_. The soil mechanical composition was analyzed using a Mastersizer 2000 Type Laser Particle Size Analyzer and the pH was analyzed by pH meter. Fire loss method [25] was used to determine the soil organic matter (SOM) content. Total nitrogen (TN), alkali-hydrolyzed nitrogen (AN), and total phosphorous (TP) were measured and the methods were described by Lu [26]. Inductively coupled plasma mass spectrometry (ICP-MS) was used to analyze Cd, Cr, Cu, Pb, and Zn concentration of soil samples [27]. Quality control and assurance were carried out by national soil standard material verification. All samples were measured in parallel two times and the relative deviation of the two parallel samples was controlled within ±10%.

### 2.3. Soil Quality Assessment Methods

#### 2.3.1. Integrated Fertility Index

Integrated fertility index (IFI) was used to evaluate soil fertility [28]:

(1) $$\mathrm{IFI}=\sum_{i}^{n}f_{i}\times a_{i},$$

where f_i_ represents the membership value of participating indexes, a_i_ is the weight of participating indexes, and n is the number of indexes.

The evaluation indexes of soil fertility were selected according to the actual situation of the study area and eight soil properties were used. The membership function included parabolic and S-type. Clay, silt, sand, and pH used the parabolic type, indicated by Equation (2). SOM, TN, AN, and TP were calculated by the S-type, indicated by Equation (3).

(2) $$f\left(x\right)=\left\{\begin{array}{c} \begin{array}{c} 0.9\times \frac{x_{4}-x}{x_{4}-x_{3}}+0.1,\;(x_{3}<x<x_{4})\\ 1.0,\;(x_{2}<x\;\leq \;x_{3}) \end{array}\\ 0.9\times \frac{x-x_{1}}{x_{2}-x_{1}}+0.1,\;(x_{1}<x\;\leq \;x_{2})\\ 0.1,\;\left(x\;<\;x_{1}\;\mathrm{or}\;x\;>\;x_{4}\right) \end{array}\right.,$$

(3) $$f\left(x\right)=\left\{\begin{array}{c} 1.0,\;\left(x\;\geq \;x_{2}\right)\\ 0.9\times \frac{x-x_{1}}{x_{2}-x_{1}}+0.1,\;(x_{1}<x\;\leq \;x_{2})\\ 0.1,(x<x_{1}) \end{array}\right..$$

The *x*_1_, *x*_2_, *x*_3_, *x*_4_ values are listed in Table 1 [21]. The weight a_i_ of participating indices was determined by the correlation coefficient method.

IFI value is positively correlated with soil fertility. The higher the IFI value, the better the soil fertility. The IFI evaluation grading standard is shown in Table 2.

#### 2.3.2. Single Factor Index and Nemerow Comprehensive Index

Soil heavy pollution is assessed by single factor index (*P_i_*) (Equation (4)) and the Nemerow comprehensive index (*P_n_*) (Equation (5)) [23]. The participating indices included Cd, Cr, Cu, Pb, and Zn.

(4) $$P_{i}=\frac{C_{i}}{S_{i}},$$

(5) $$P_{n}=\sqrt{\frac{{\left(\bar{P}\right)}^{2}+P_{imax}{}^{2}}{2}},$$

where *C*_i_ represents the concentration of heavy metal, and S_i_ is the background value of heavy metal in Jiangxi province. $\bar{P}$ is the arithmetic mean value of *P_i_* of total heavy metals in one sampling site, and $P_{imax}$ represents the maximum of *P_i_*. The classification standard of the evaluation index is listed in Table 3.

#### 2.3.3. Potential Ecological Risk Index

The evaluation method of potential ecological risk index proposed by Hakanson [29] not only considers the content of heavy metals, but also comprehensively considers the synergistic effect of various elements, toxicity level, and environmental sensitivity to heavy metal pollution, etc. Therefore, it is widely used to evaluate the potential ecological risk in soil environment, and its calculation formula is as follows:

(6) $$\mathrm{RI}=\sum_{i=1}^{n}E_{r}^{i}=\sum_{i=1}^{n}(T_{r}^{i}\times C_{i}/C_{n}^{i}),$$

where RI represents the total potential ecological risk index for all heavy metals, $E_{r}^{i}$ is the single factor potential ecological risk index of heavy metal i. $T_{r}^{i}$ is the toxicity coefficient of heavy metal i, $C_{i}$ represents the content of heavy metal i in soil (mg/kg), and $C_{n}^{i}$ is the background value of heavy metal i in Jiangxi province. $T_{r}^{i}$ of Cd, Cr, Cu, Pb, and Zn are 30, 2, 5, 5, and 1, respectively. The classification standard of the evaluation index is listed in Table 4.

#### 2.3.4. Comprehensive Assessment of Soil Quality

Based on the positive contribution of soil fertility to comprehensive soil quality and the negative impact of soil heavy metals on comprehensive soil quality, combined IFI with *P_n_*, the TOPSIS method was proposed to evaluate the comprehensive soil quality. The basic principle of the TOPSIS method is to determine a positive ideal point and a negative ideal point for each evaluation index [30]. The distance between each evaluation object and the positive ideal point and the negative ideal point was calculated by the Euclidean distance method using the evaluation index, and the distance measures the merits and demerits of the evaluation object. The steps are as follows:

Step 1. Forward processing: the Nemerow comprehensive index in this study is a very small index, that is, the smaller the index is, the higher the soil quality is. Therefore, the Nemerow comprehensive index should be forward processed, and the calculation formula is as follows (the IFI value does not need to do forward processing treatment):

(7) $$x_{ij}=x_{max}-x,$$

where $x_{max}$ is the max value of the Nemerow comprehensive index of all samples, $x_{ij}$ is the index after forward processing, and x is the index before forward processing.

Step 2. Index standardization treatment: the forward matrix $x^{\prime }$ obtained after forward processing:

(8) $$X^{\prime }=\left[\begin{array}{ccc} x_{11}^{\prime } & \cdots & x_{1m}^{\prime }\\ \vdots & \ddots & \vdots \\ x_{n1}^{\prime } & \cdots & x_{nm}^{\prime } \end{array}\right],$$

where *n* is the number of evaluation objects, and m is the number of evaluation indexes of each object. After standardization, the standardized matrix *Z* can be obtained:

(9) $$Z=\left[\begin{array}{ccc} z_{11} & \cdots & z_{1m}\\ \vdots & \ddots & \vdots \\ z_{n1} & \cdots & z_{nm} \end{array}\right].$$

The matrix standardization formula is as follows:

(10) $$z_{ij}=x_{ij}^{\prime }/\sqrt{\sum_{i=1}^{n}x_{ij}^{\prime 2}},$$

where $z_{ij}$ represents index j of object i after standardization.

Step 3. determine the positive ideal (*Z^+^*) and negative ideal (*Z^−^*) solutions for each criterion:

(11) $$Z^{+}=\;\left(max\left\{z_{11},\ldots ,z_{n1}\right\},\ldots ,max\left\{z_{1m},\ldots ,z_{nm}\right\}\right),$$

(12) $$Z^{-}=\;\left(min\left\{z_{11},\ldots ,z_{n1}\right\},\ldots ,min\left\{z_{1m},\ldots ,z_{nm}\right\}\right).$$

Step 4. Calculate the distance using the n-dimensional euclidean distance.

(13) $$D_{i}^{+}=\sqrt{\sum_{j}^{m}{\left(Z_{j}^{+}-z_{ij}\right)}^{2}},$$

(14) $$D_{i}^{-}=\sqrt{\sum_{j}^{m}{\left(Z_{j}^{-}-z_{ij}\right)}^{2}}.$$

In the formula, $D_{i}^{+}$ and $D_{i}^{-}$ are the distances from the evaluation object *i* to the positive ideal point and to the negative ideal point, respectively.

Step 5. Calculate the relative closeness to the ideal solution. The calculation formula is as follows:

(15) $$S_{i}=\frac{D_{i}^{-}}{D_{i}^{-}+D_{i}^{+}},$$

where *S_i_* is the comprehensive evaluation index of soil quality, and the larger the value, the higher the comprehensive quality level of soil.

### 2.4. Data Analysis

Statistical analysis was conducted using ArcGIS 10.2 (ESRI, Redlands, CA, USA), SPSS 22.0 (SPSS Inc., Chicago, IL, USA) and R 4.1.1 (R Core Team, 2021, Vienna, Austria). Furthermore, the sampling sites, soil fertility grades, soil heavy metal pollution grades, and comprehensive spatial distribution of soil quality were drawn by ArcGIS 10.2. Inverse distance weighing (IDW) method was used to conduct spatial interpolation. Pearson correlation analysis was analyzed by SPSS 22.0 and R 4.1.1. Source apportionment of soil pollutant was analyzed by PMF 5.0 (USEPA, Washington, DC, USA).

## 3. Results and Discussion

### 3.1. Soil Indicators Characteristics and Summary of Contamination

**Figure 2:**
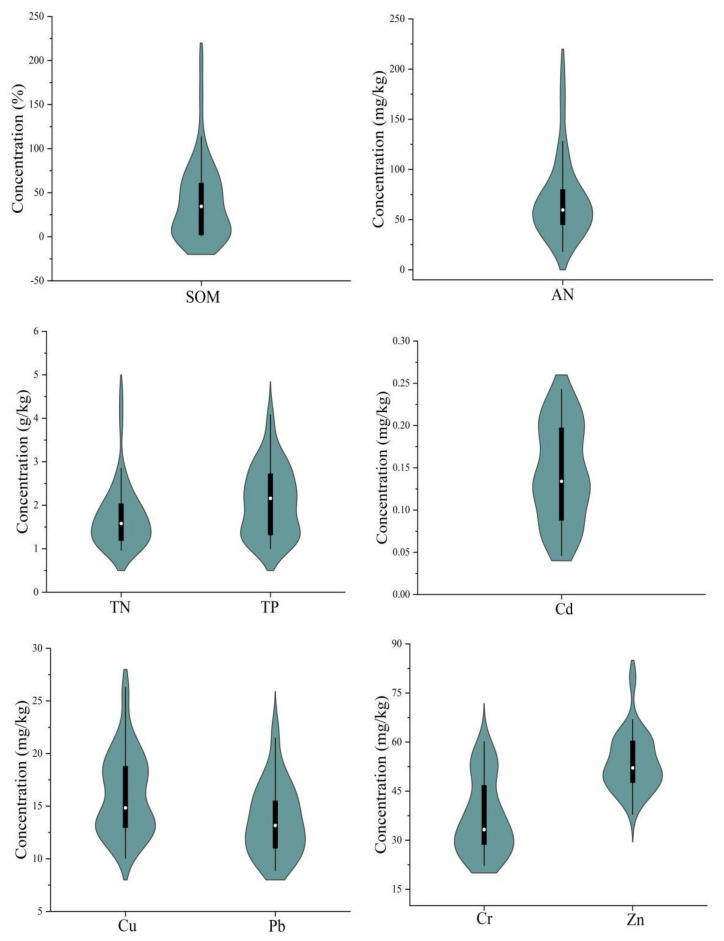
Violin of soil property concentrations (n = 43). The white point represents the median value of concentration. The black boxes range from the lower quartile to the upper quartile. The tentacles extend to the most extreme data point, which do not exceed 1.5 times the IQR (interquartile spacing) of the boxes.

The physicochemical properties of the soil and the descriptive statistics in the study area are shown in Table 5 and Figure 2. There were no notable differences in the mechanical composition between different land uses. All the samples of average silt were more than 50%, which showed that the soil texture was loam. The pH value ranged from 4.28 to 4.99, which showed strongly acidic soil. Among them, grassland soil was lower than 4.5. The mean values of SOM under different land uses occurred in the following order: dry land > orchard > forest > vegetable > paddy > grassland. According to the standard classification of soil nutrients in the Second National Soil Census, only SOM in dry land soils was at the second level, and the remaining soil types were all at the third (medium) level. A previous study reported that SOM in rhizosphere soil increased significantly after paddy was transformed into vegetable [31]. The mean values of TN followed the sequence: dry land > vegetable > grassland > orchard > forest > paddy. TN and AN in dry land and vegetable were at the first (rich) level and third level, respectively. AN in remaining soils were below the fourth (poor) level. TP in all soil types were at the first level. The average nutrient contents of all soil types were SOM 2.34%, TP 2.08 g/kg, TN 1.73 g/kg, AN 69.68 mg/kg. These results showed that soil fertility quality was generally not optimistic in the study area.

As Table 5 showed, the heavy metal concentration mean value in all soil types occurred in the following order (mg/kg): Zn (53.80) > Cr (36.92) > Cu (16.14) > Pb (13.68) > Cd (0.14). In particular, the mean value of Cd was higher than the background value of Jiangxi province and Beijing [32,33], especially two times higher than China and Spokane, USA [32,34], which might be related to mining ionic rare earth minerals [35]. Cd and Cu were lower than Beijing, but higher than Spokane, USA. Pb and Zn were lower than other study areas [32,33,34]. Moreover, the coefficient of variation (CV) of heavy metals were as follows: Cd (0.40) > Cr (0.31) > Pb (0.25) > Cu (0.24) > Zn (0.17). The high CV values of Cd, Cr and the moderate CV values of Pb, Cu indicated that anthropogenic activities or high geological background might influence their distribution. As for Cd, Cr, Cu and Pb, orchard soils ranked first. Forest soils ranked first in terms of Zn. Overall, the contents of these heavy metals showed large variances between different land use types, possibly on account of the nature of various crops.

**Table 5:** The descriptive statistics of physicochemical properties in soil samples.

			Clay	Silt	Sand	pH	SOM	TN	AN	TP	Cd	Cr	Cu	Pb	Zn
			%				%	g/kg	mg/kg	g/kg	mg/kg				
Min			3.36	45.61	24.46	4.15	1.18	0.97	18.25	1.00	0.05	22.28	10.04	8.87	37.90
Max			8.38	67.16	48.27	5.98	4.62	4.53	203.05	4.08	0.24	60.12	26.28	22.64	80.15
Mean			5.91	57.71	36.38	4.75	2.34	1.73	69.68	2.08	0.14	36.92	16.14	13.68	53.80
Standard Deviation			1.11	5.79	5.85	0.51	0.78	0.74	39.62	0.79	0.06	11.38	3.89	3.37	8.94
Coefficient of Variation			0.19	0.10	0.16	0.11	0.33	0.43	0.57	0.38	0.40	0.31	0.24	0.25	0.17
BG_1_ ^a^											0.10	48.00	20.80	32.10	69.00
BG_2_ ^b^											0.07	53.90	20.00	23.60	67.70
Other cities															
Beijing, China [33]											0.13	56.00	27.30	26.90	78.30
Spokane, Washington, USA [34]											0.07	25.80	12.50	19.00	54.00

^a^ Natural background values of soil heavy metals in Jiangxi Province [32]. ^b^ Natural background values of soil heavy metals in China [32].

### 3.2. Correlational Analysis between Different Soil Indicators

**Figure 3:**
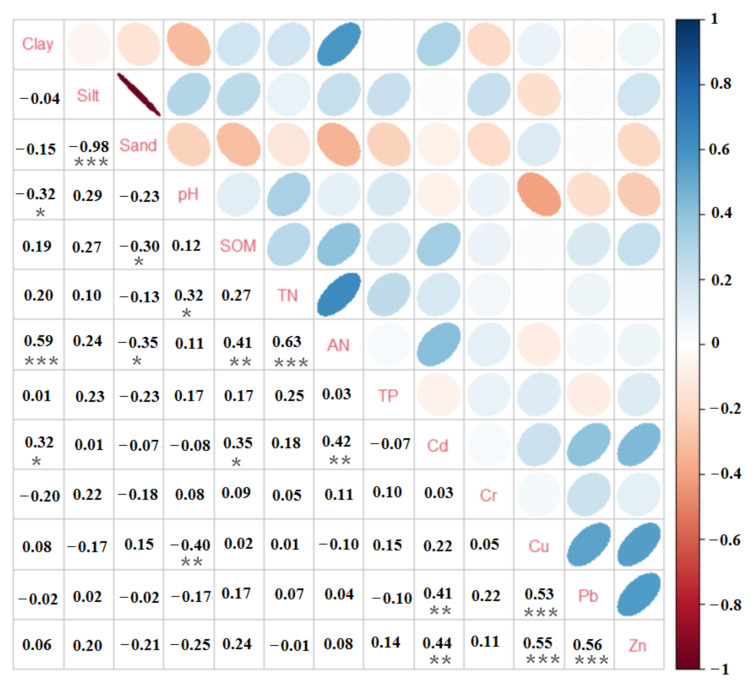
Correlation matrix of soil properties. The number indicate strong correlation (* *p* < 0.1 and ** *p* < 0.05) or significant correlation (*** *p* < 0.01).

As shown in Figure 3, clay and AN (0.59), TN and AN (0.63), Cu and Pb (0.53), Cu and Zn (0.55), Pb and Zn (0.56) all showed strong positive correlation. The correlations between SOM and AN, AN and Cd, Cd and Pb, and Cd and Zn were positive (0.4–0.5). The correlation between silt and sand (−0.98) was strong negative. Moreover, pH and Cu, pH and clay, SOM and sand, and sand and AN all had negative correlations. The significant correlations between different heavy metals (Cu and Pb, Cu and Zn, Pb and Zn, Cd and Pb, and Cd and Zn) showed that soil was more likely to be contaminated by multiple heavy metals at the same time [36,37]. From Figure 3, it can be learned that chemical properties mainly showed significant correlations. The underlying reason was that various human activities contributed to the changes under different land uses in the study area [38].

### 3.3. Soil Fertility Assessment

**Figure 4:**
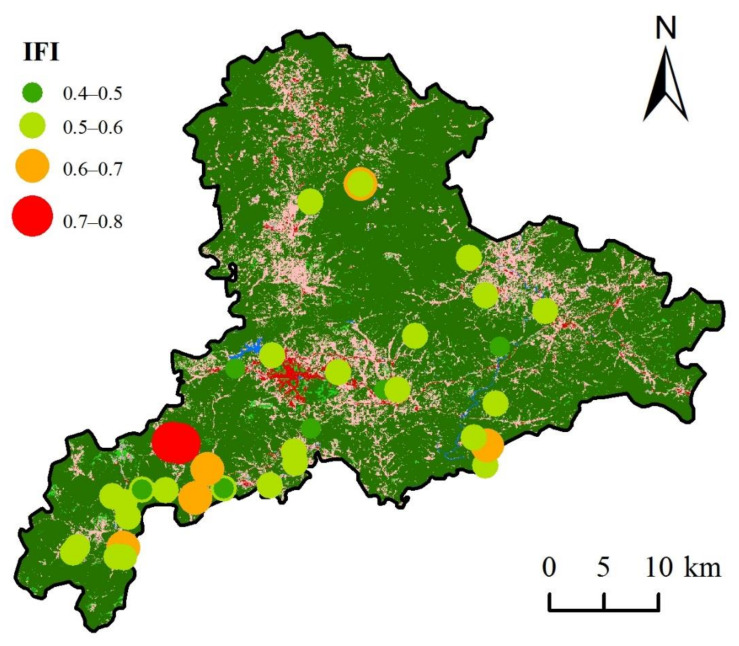
Spatial distribution of integrated fertility index (IFI) in the study area.

According to the assessment results, the soil samples had 23.26% at Level IV (low soil quality), 60.47% at Level III, 11.63% at Level II and 4.64% at Level I (high soil quality). The soil includes six types in the study area: vegetable soil, orchard soil, paddy soil, forest soil, dry land soil, and grassland soil. As indicated, vegetable soil samples had 20% of its quality at Level IV, 60% at Level III, and 20% at Level I. Orchard soil and forest soil samples were Level III and Level II, and most of these two kinds of soil were Level III. Paddy soil samples had 55% at Level IV and 45% at Level III. The mean IFI values were ranked as follows: dry land soil > vegetable soil > orchard soil > grassland soil > forest soil > paddy soil. It can be learned that vegetable soil was the best, with paddy soil being the worst. Since soil pH and SOM content depend highly on land use and cropping pattern (e.g., paddy land, vegetable land, and abandoned land) [39], it is reasonable to get different IFI values under different land type. A previous study reported that average SOM contents in different land uses ranked as follows: paddy field > abandoned cropland > vegetable land [40,41]. Figure 4 showed that the overall fertility quality was at a low level and the western study area was the high value region.

### 3.4. Soil Heavy Metal Pollution Assessment and Ecological Risk Assessment

Single factor index (*P_i_*) and Nemerow comprehensive index (*P_n_*) were calculated, and the results are shown in Table 6 and Figure S1. The average *P_i_* values of Cr, Cu, Pb, and Zn were all lower than 1 and the average value of Cd was 1.43, which meant that the study area was generally safe. The maximum *P_i_* of Cd, Cr, and Zn were 2.43, 1.25, and 1.16, respectively and the maximum value of *P_n_* was 1.85, which was larger than 0.7. This indicated that point source pollution might exist to some extent in the study area. According to the grading standards of the evaluation index in Table 3, *P_i_* of Cd showed that 32 sampling points exceeded the standard including degrees II and III, but Cd, Cu, and Zn sampling points exceeding the risk index were all at degree II. The *P_n_* showed that 30 sampling points exceeded the standard and the over-standard rate was 70%, further proving that there were a few points with pollution risks, and it was necessary to pay attention to the pollution points. *P_i_* of Cd, Cr, Cu, and Pb under orchard land use ranked first, and *P_i_* of Cd, Cr, Pb, and Zn under grassland were the lowest. Some studies have shown that in terms of soil heavy metal pollution, the difference between the less disturbed areas (e.g., grassland and wasteland) and disturbed areas (e.g., garden and tailing) may be due to the slow release of metals from minerals from geological sources with low availability [42,43].

**Figure 5:**
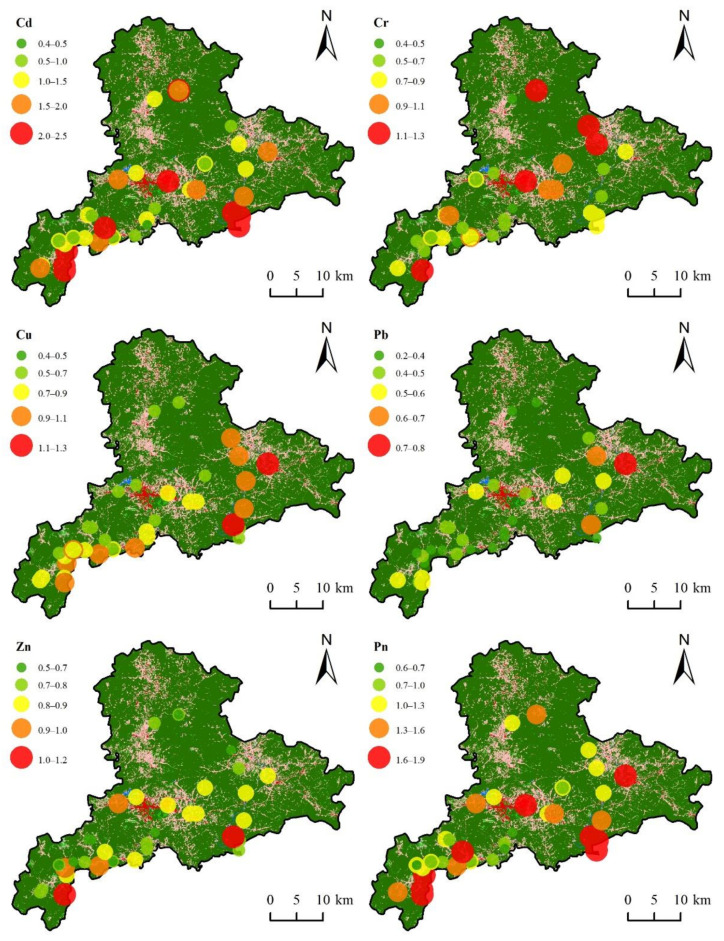
Spatial distribution of single factor index (*P*_i_) and Nemerow comprehensive index (*P_n_*) in the study area.

The spatial distributions of *P_i_* and *P_n_* of soil heavy metals are shown in Figure 5. The *P_i_* distribution of Cu, and Pb showed a gradual increase from west to east, and the high values of Cr were distributed in the northeast. The spatial distribution of *P_n_* was similar to *P_i_* of Cd, because *P_n_* highlights the impact and effect of the pollutants with the largest *P_i_* on environmental quality. The heavy metal pollution with the most serious pollution degree is highlighted, resulting in a large proportion of Cd in *P_n_*. Heavy metals in urban soil are mainly related to human activities, such as heavy metal dust and garbage from human sources such as domestic construction waste and automobile exhaust and the content of soil heavy metals can be increased by dry and wet settlement or stacking [44,45]. This study found that the sample points with *P_n_* larger than 1 were distributed in the eastern regions. From land use factors, the regions were densely populated with population, traffic, and commerce, and the main traffic arteries and bus stations were mainly distributed in this region. There was no large area pollution in the study area, and only a small part of the area with light pollution.

The ecological risk assessment of soil heavy metals was shown in Table 7. $E_{r}^{i}$ of Cr, Cu, Pb, and Zn were at a slight level, with an average value lower than 40. However, the $E_{r}^{i}$ of Cd was 13.65~73.05, with an average value of 42.75 and 51% soil samples at a moderate level. The value range of RI was 20.53~79.94, with the average value of 51.08. All the samples were at slight level in terms of RI. Among all the detected heavy metals, Cd had the highest contribution rate to RI (83.70%), followed by Cu and Pb (7.60% and 4.17%). Therefore, Cd is the main potential ecological risk heavy metal element in the soil of the study area. Because Cd has good activity, strong migration ability, and is easily absorbed by plants, it is toxic to almost all organisms [46]. Therefore, a higher content of Cd in soil will lead to higher ecological risk.

### 3.5. Spatial Distribution of Comprehensive Soil Quality

**Figure 6:**
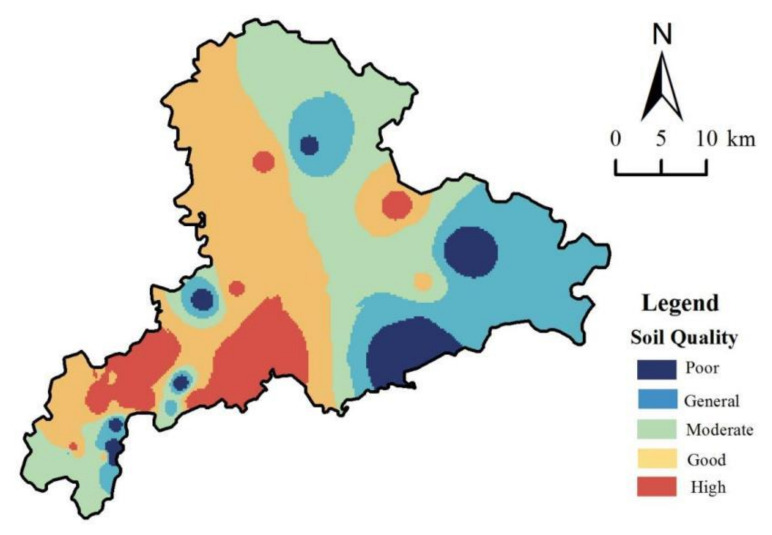
Spatial distribution of comprehensive soil quality.

TOPSIS was used to calculate the comprehensive soil quality index, and ArcGIS natural breakpoint method can classify the comprehensive soil quality. A total of 38 samples were used to draw the spatial distribution map and 5 samples were used to verify the accuracy and precision. The results showed that accuracy is −0.02 (normalized mean error) and the precision is 0.239 (normalized root mean square error). According to the research results, the study area was divided into five types: high quality area, good quality area, moderate quality area, general quality area, and poor quality area. The study area was 10.9% at high quality, 31.7% at good quality, 29.0% at moderate quality, 22.0% at general quality, and 6.4% at poor quality. The high quality area was situated in the western of the study area (Figure 6) in which *P_n_* was lower and soil fertility was higher. The contents of SOM, TN, AN, and TP were higher than those in other regions, and there was no soil heavy metals pollution. The indexes of the poor region and the general region were far from the ideal target, indicating that the soil fertility level of the region was low or there was potential pollution risk, which was mainly distributed in the eastern of the study area.

From the perspective of different land use types, the TOPSIS values were ranked as follows: vegetable soil > paddy soil > forest soil > dry land soil > grassland soil > orchard soil.

### 3.6. Source Apportionment of Soil Pollutant

**Figure 7:**
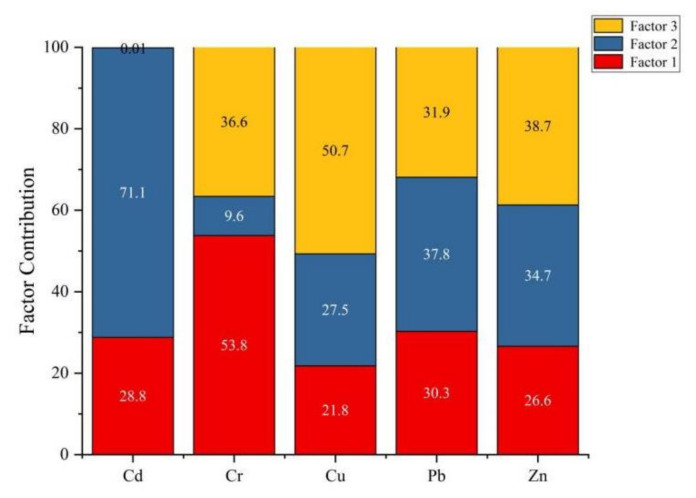
Analytical contribution of heavy metal PMF source.

As shown in Figure 7, in terms of Cd and Pb, the contribution rate of factor 2 was relatively high with 71.1% and 37.8%, respectively. Pb is the main indicator of transport emissions because it comes from vehicle fuel burning, vehicle engine, and tire friction [47]. Cd exists in car exhaust. These pollutants can be accumulated through atmospheric sedimentation and dust adsorption ways, thus causing pollution to the cultivated soil [48,49,50]. Highway, national road, and provincial road run through the study area, and heavy metal elements in the tail gas emitted by vehicles accumulate in the soil through atmospheric deposition and air adsorption, thus causing pollution. Hence, factor 2 can be regarded as transportation source. The contribution rate of factor 1 to Cr was 53.8%. It is generally believed that the main source of Cr is soil parent material [51,52]. The statistics of soil heavy metal content showed that the average content of Cr in soil were lower than the background value of Cr in Jiangxi Province. Therefore, factor 1 is natural source. The contribution rate of factor 3 to Cu and Zn was 50.7%. and 38.7%, respectively. Cu and Zn are important components of pesticide and fertilizer [53,54]. The soil types of the study area are forest, paddy field, and orchard. The above analysis showed that the average content of Cu in soil of orchard was the highest and Zn in forest was the highest, respectively. Therefore, factor 3 is the agriculture source.

## 4. Conclusions

In this study, we assessed the soil quality in a study area with different land uses. The results showed that the AN content was low, and SOM, TP, and TN were rich. The overall soil fertility level was medium and lower. The mean IFI values were ranked as follows: dry land soil > vegetable soil > orchard soil > grassland soil > forest soil > paddy soil. The risk of soil heavy metal pollution in a few areas had reached the warning line and slight pollution line, and there was a risk of potential pollution. *P_i_* of Cd, Cr, Cu, and Pb under orchard land use ranked first, and *P_i_* of Cd, Cr, Pb, and Zn under grassland were the lowest. Cd was the main pollutant in the soil of the study area, which had strong ecological risk. Cd should be paid more attention to, and protection measures should be taken. TOPSIS evaluation results showed that the comprehensive soil quality was mainly of good and moderate quality, accounting for 31.7% and 29.0% of the total agricultural land area, respectively. The PMF model results showed that the transportation source contributes a lot in terms of Cd and Pb. As for Cr, the natural source contributes 53.8%. In terms of Cu and Zn, the agriculture source contributes 50.7% and 38.7%, respectively. The study revealed the differences of soil quality among different land use types in the red soil region and the study results are important evidence for scientifically managing and reasonably using the soil resources.

## Figures and Tables

**Table 1:** The turning points of membership function of soil fertility indexes.

Turning Point	Clay %	Silt %	Sand %	pH	SOM %	TN g/kg	AN mg/kg	TP g/kg
X1	20	20	20	6.0	3.5	0.4	40	0.4
X2	40	40	40	7.5	5	2	120	1.5
X3	60	60	60	8.0				
X4	80	80	80	9.0				

**Table 2:** The classification criterion of soil integrated fertility index (IFI).

Fertility Degree	I	II	III	IV
IFI	IFI > 0.7	0.7 ≥ IFI > 0.6	0.6 ≥ IFI > 0.5	0.5 ≥ IFI

**Table 3:** The classification criterion of evaluation index.

Degree	Singe Factor Index Method		Nemerow Comprehensive Index Method	
	Single Factor Index (*P_i_*)	Pollution Level	Nemerow Comprehensive Index (*P_n_*)	Pollution Level
I	*P_i_* ≤ 1	None	*P_n_* ≤ 0.7	Safe
II	1 < *P_i_* ≤ 2	Light	1 < *P_n_* ≤ 2	Warning line
III	2 < *P_i_* ≤ 3	Moderate	2 < *P_n_* ≤ 3	Light
IV	3 < *P_i_*	Serious	2 < *P_n_* ≤3	Moderate
V	—	—	3 < *P_n_*	Serious

**Table 4:** The classification criterion of potential ecological risk index.

$E_{r}^{i}$	Level	RI	Level
$E_{r}^{i}$ < 40	Slight	RI < 150	Slight
$40\;\leq \;E_{r}^{i}$ < 80	Moderate	150 ≤ RI < 300	Moderate
$80\;\leq \;E_{r}^{i}$ < 160	Strong	300 ≤ RI < 600	Strong
$160\;\leq \;E_{r}^{i}$ < 320	Serious	600 ≤ RI < 1200	Serious
$E_{r}^{i}\;$≥ 320	Very serious	RI ≥ 1200	Very serious

**Table 6:** The descriptive statistics of heavy metal evaluation index.

Evaluation	Soil Index	Minimum	Maximum	Average	Standard Deviation
Single factor index	Cd	0.46	2.43	1.43	0.57
	Cr	0.46	1.25	0.77	0.24
	Cu	0.48	0.26	0.78	0.19
	Pb	0.28	0.71	0.43	0.11
	Zn	0.55	1.16	0.78	0.13
Nemerow comprehensive index		0.64	1.85	1.20	0.37

**Table 7:** Evaluation results of potential ecological risk of soil heavy metals.

Evaluation	Soil Index	Value Range	Average	Number of Samples at Each Level				
				Slight	Moderate	Strong	Serious	Very Serious
$E_{r}^{i}$	Cd	13.65~73.05	42.75	21	22	0	0	0
	Cr	0.93~2.51	1.54	43	0	0	0	0
	Cu	2.41~6.32	3.88	43	0	0	0	0
	Pb	1.38~3.53	2.13	43	0	0	0	0
	Zn	0.55~1.61	0.78	43	0	0	0	0
RI		20.53~79.94	51.08	43	0	0	0	0

## Data Availability

Data supporting this study are available to the principal investigator upon request.

## Supplementary Materials

The following supporting information can be downloaded at: https://www.mdpi.com/article/10.3390/ijerph19074125/s1, Figure S1: Violin of single factor index and nemerow comprehensive index (n = 43).
