# Supplementary material for: Heavy Metal Pollution and Soil Quality Assessment under Different Land Uses in the Red Soil Region, Southern China
Source: Int J Environ Res Public Health. 2022 Mar 31;19(7):4125. doi: 10.3390/ijerph19074125 (PMC8998205; doi:10.3390/ijerph19074125)
Supplement: Supplementary file 1 [file ijerph-19-04125-s001.zip › Supplementary Materials.pdf]

Supplementary Materials:

Heavy metal pollution and soil quality assessment under different land uses in the red soil region, southern China

Zhiping Yang [1], Rong Zhang [2], Hongying Li [3], Xiaoyuan Zhao [4] and Xiaojie Liu [4,*]

[1] Jiangxi Research Academy of Ecological Civilization, Nanchang 330036, China

[2] Technical Centre for Soil, Agriculture and Rural Ecology and Environment, Ministry of Ecology and Environment, Beijing 100012, China

[3] Foreign Environmental Cooperation Center, Ministry of Ecology and Environment, Beijing 100035, China

[4] Institute of Geographical Sciences and Natural Resources Research, Chinese Academy of Sciences, Beijing 100101, China

** Corresponding author: liuxj@igsnrr.ac.cn*

Figure listed:

Figure S1. Violin of single factor index and nemerow comprehensive index (n = 43). White point represents the median value. Black boxes range from the lower quartile to the upper quartile. Tentacles extend to the most extreme data point, which do not exceed 1.5 times the IQR (interquartile spacing) of the boxes.

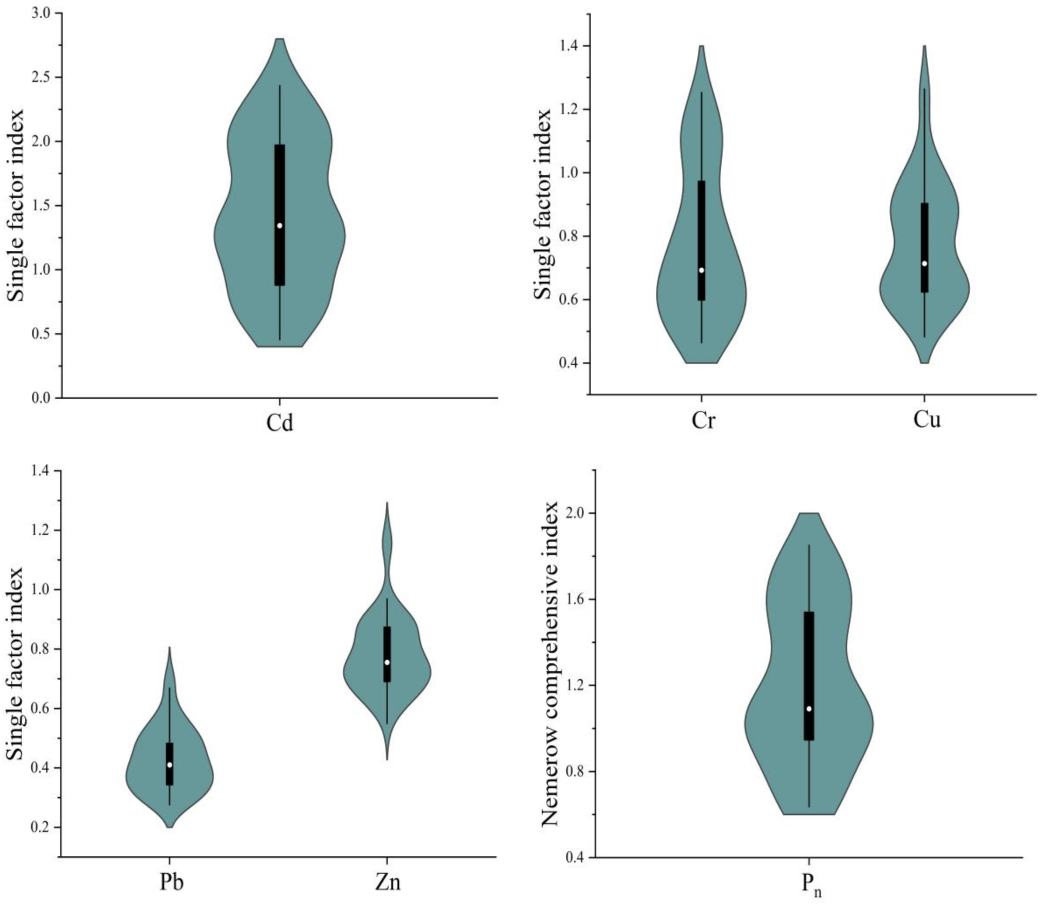

Figure S1. Violin of single factor index and nemerow comprehensive index (n = 43). White point represents the median value. Black boxes range from the lower quartile to the upper quartile. Tentacles extend to the most extreme data point, which do not exceed 1.5 times the IQR (interquartile spacing) of the boxes.
